# The joint effect of personality traits and perceived stress on pedestrian behavior in a Chinese sample

**DOI:** 10.1371/journal.pone.0188153

**Published:** 2017-11-30

**Authors:** Tingting Zheng, Weina Qu, Yan Ge, Xianghong Sun, Kan Zhang

**Affiliations:** 1 CAS Key Laboratory of Behavioral Science, Institute of Psychology, Beijing, China; 2 Department of Psychology, University of Chinese Academy of Sciences, Beijing, China; Beihang University, CHINA

## Abstract

While improper pedestrian behavior has become an important factor related to road traffic fatalities, especially in developing countries, the effects of personality traits and/or stress on pedestrian behavior have been rarely reported. The current study explored the joint effects of five personality traits (i.e., extraversion, openness, neuroticism, normlessness and altruism) and global perceived stress (measured with the Perceived Stress Scale-10) on pedestrian behavior (measured with the Pedestrian Behavior Scale) in 311 Chinese individuals. Results showed that altruism, neuroticism and openness significantly affected different pedestrian behavior dimensions, while global perceived stress also significantly and positively predicted positive behavior. Moreover, the effect of neuroticism on positive behavior was fully mediated by stress. Some explanations and implications are provided in the discussion section.

## Introduction

As a group of main road users, pedestrians are subject to few rules, which results in widespread illegal or improper behavior [1]. Pedestrian violations have become a serious social problem [2]. Keegan and O’Mahony [3] reported that 35% of pedestrians enter illegally at a signalized crossing. Besides illegal red light walking, other likely causes of pedestrian-involved accidents include illegal pedestrian movements, inattentiveness, negligence, inappropriate management, and lack of reasonable facilities to cross streets [1, 4, 5]. It was demonstrated that one of the “predominant contributing factors” of pedestrian crashes is violation of traffic laws by the victim in El Paso County, Texas [6]. To better understand the causes of traffic accidents and reduce them, it’s pertinent to assess pedestrian behavior.

While pedestrian-involved traffic accidents have become a major safety problem worldwide, it is especially serious in developing countries, due to high population density, rapid urbanization, and poor performance of road users in traffic regulation adherence [7]. The percentage of road traffic deaths among pedestrians is estimated to range from 11% in the very low-mortality sub-region of the Americas to 55% in the high-mortality African sub-region [8]. It is appalling that 78% of overall traffic fatalities in Peru are pedestrians, while Mozambique and El Salvador both show rates above 60% (all the three countries are low-income countries) [9]. In China, a developing country with increasingly crowed urban traffic and 40% pedestrians in big cities [10], issues regarding pedestrian injuries and fatalities are particularly serious. Indeed, Chinese pedestrians are the most vulnerable road users, suffering from vehicle-pedestrian accidents more frequently [11]. While traffic-related fatalities continued to be the leading cause of injury and death (12.45 per 100,000 individuals) in 2011 [12–14], about one quarter of all mortalities were pedestrians [15]. China also had the highest absolute number of pedestrian deaths in the world in 2009 [16]. Considering that Chinese pedestrian awareness of adherence to traffic regulations is relatively weak, and the unique effects of economic and social development on traffic environments, there is an urgent need to explore pedestrian behavior in China.

Researches about pedestrian behavior have been carried out for years, with various approaches developed to measure pedestrian behavior, including field study (unobtrusive observation or video record) [17, 18], simulation study [19], building mathematical models [20], and self-reported questionnaires [21, 22]. The Pedestrian Behavior Scale (PBS) is a self-reported scale to assess injury risk behaviors among pedestrians of all ages, and was developed by Granié et al. [21]. It was compiled using the conceptual framework of the Driver Behavior Questionnaire [23], scales of aggressive driver behaviors [24] and positive driver behaviors [25] toward other road users. A four-factor structure was constructed, involving intentional transgressions concerning offenses and errors, unintentional lapses related to lack of concentration, aggressive behaviors referring to the expression of negative emotions and aggressive interactions, and positive behaviors comprising conciliatory social interactions. It was recently validated in China, and the Chinese version has acceptable internal reliabilities [26].

Personality traits is associated with the level of acceptable risk which one is willing to assume, thus may reflect risky behaviors among pedestrians. With the lack of systematic researches correlating various personality traits with pedestrian behaviors, results from driver studies provide some references. In previous reports, personality variables such as normlessness [27], anger [28, 29], sensation seeking [30], altruism and the Big Five personality factors [31–34] generally affect driving behaviors in the road traffic system. We hypothesized that these traits may also affect pedestrian behaviors. For example, normlessness refers to individual’s disrespect for adherence to norms [35]. Pedestrians who are more normless than others (i.e., less respectful for adherence to norms) may have more transgressive and aggressive behaviors, just like normless drivers are more likely to violate rules and drive with high risk [27]. This hypothesis was partly proven in one of our recent work [26]. We explored the associations of normlessness, altruism, anger, and sensation-seeking with pedestrian behavior in Chinese individuals. Pedestrian behavior was measured with the Chinese version of the Pedestrian Behavior Scale [21]. The results indicated that normlessness is a significant predictor of transgressive and aggressive behaviors, probably because people with high normlessness scores demonstrate a negative attitude towards traffic rules and perform risk-taking behaviors more frequently [36]. Meanwhile, individuals who scored higher in altruism (i.e., more concerned about others) showed more positive behaviors and less frequent lapses. Anger was also related to more aggressive behaviors.

Nevertheless, the effects of other personality traits such as the Big Five [37] on pedestrian behaviors were barely noticed. As the most well-established and widely used conceptual models of personality, the Big Five Factor Model may help understand individual differences in pedestrian behavior more deeply. As known, openness to experience is associated with intellectual curiosity, creativity, and a preference for novelty and variety. Extraversion is associated with significant engagement, assertiveness, and sociability [38]. Both openness and extraversion have been theoretically and empirically linked to sensation seeking [39–42], which has been found to positively correlated to the amount of hit suffered in a virtual pedestrian environment [43]. However, a study by Herrero–Fernández et al. [22] found no direct relationship between openness and risk-taking among young adult pedestrians. This result needs to be verified in a larger sample, and also in developing countries. Neuroticism is the tendency to experience unpleasant emotions easily and is sometimes referred to as emotional instability, and may also affect pedestrian behavior. Neuroticism and extraversion have been associated with work accident involvement [44], increased road rage [45] and aggressive driving [46]. This study aimed to assess the utility of combining five personality traits (i.e., normlessness, altruism, extraversion, openness and neuroticism) in predicting self-reported pedestrian behaviors, while such personality traits have been usually studied in isolation [22, 43].

Another factor concerned in this study was stress. Nowadays, stress seems to be a problem faced by nearly everyone. It is reasonable to suspect that stress may do harm to pedestrian behaviors, given that it has been revealed to affect attention [47], working memory [48], perceptual-motor performance [49] and anxiety [50]. Previous studies assessing driving safety have revealed the hazardous effect of perceived stress on driving behavior. The influencing factors included not only driving-related stress [51–54], but also the global level of stress [55], a type of subjective, perceived psychological stress which primarily arises from the overall feeling of daily life. For instance, one may perceive high level of global stress when life has become unpredictable, out of control, or overwhelming [55]. Global stress doesn’t emphasize any solo stressor, but covers the effects of all potential stressors. Considering that walking doesn’t need special skills like driving, and thus may arouse little walking-related stress itself, global perceived stress, mainly caused by everyday stressors such as life events, work and daily frustrations, is much more likely to affect pedestrian behavior.

Furthermore, we hypothesized that stress may play a transitive role in the effects of personality traits on pedestrian behavior. Personality may affect how stressors are experienced, including frequency, intensity, and nature of stressors [56]. For example, neuroticism is generally associated with high rates of stress exposure and impaired ability to cope with stress [57–59]. Schneider et al [60] discovered that positive aspects of personality, namely extraversion and openness, also affect stress response. Personality factors may also explain, in part, individual differences in stressor-related affect. For example, people who have higher levels of neuroticism reported higher levels of negative affect on days stressors occur [61, 62], while higher levels of extraversion, conscientiousness, and openness to experience were related to less stressor-related negative affect [62]. Agreeableness was associated with decrease in stressor-related positive affect [62]. In traffic safety, neuroticism also predicts reported driver stress among drivers whose circadian type was morningness [63]. Thus, personality traits may also influence the way pedestrians perceive daily stressors and their stressor-related affect, subsequently affecting their behaviors on the road and how they cope with traffic affairs.

The main aims of the current study were as follows:

1. To assess the associations of personality traits, including extraversion, openness, neuroticism, normlessness and altruism with pedestrian behavior;
2. To evaluate the relations between the global perceived stress of pedestrians and pedestrian behavior;
3. To examine the potential mediating effect of global perceived stress on the associations of each personality traits with pedestrian behavior.

## Methods

### Participants

This survey was conducted in Anshun, Guizhou, as a part of a project named “Urbanization Project”, which was aimed at knowing the overall situation of the local residents’ living and travelling after an urbanization process. Participants were recruited from public places, including office buildings, local residences, corner shops and supermarket gates in Anshun. They agreed to participate in the study and completed a set of questionnaires voluntarily and anonymously. In total, 409 respondents returned their questionnaires. 98 questionnaires were eliminated from further analysis for the following reasons: surveys lacking an excessive amount of data; respondent skipping some items regularly; same score provided for all the items; overt pattern in the answers; selection of contradictory options in consistent or opposite descriptions, occurring more than 5 times. Ultimately, a total of 311 respondents, including 143 males (45.98%, gender of two participants were unknown), were included. They ranged in age from 16 to 75 years old (*M* = 33.64, *SD* = 11.59).

### Measures

#### Personality scales

Five personality traits were measured: Extraversion, Openness, Neuroticism, Normlessness and Altruism. The first three traits were assessed using scales from Big Five Inventory-44 (BFI-44) [64] (see S1 Appendix). All three scales showed impressive reliabilities with *α* = .88 for Extraversion (8 items), *α* = .81 for Openness (10 items) and *α* = .84 for Neuroticism (8 items) [64]. Normlessness is a trait referring to the belief that socially unacceptable behaviors are required to achieve certain goals. It was measured using the Kohn and Schooler’s [35] normlessness scale (4 items) (see S2 Appendix), for which the Cronbach’s alpha coefficient was reported to be .71 [36]. Altruism refers to an individual’s propensity to be cooperative, kind hearted, and actively concerned about others. It was measured using the facet of a Big Five personality factor: agreeableness (see S3 Appendix). All the 10 items for the altruism scale were adopted from the International Personality Item Pool (IPIP, available at http://ipip.ori.org), with a Cronbach’s alpha coefficient of .73 [65]. Each item of the five personality scales was answered on a 5-point Likert scale, ranging from 1 (“strongly disagree”) to 5 (“strongly agree”). The total score of each scale was obtained by adding scores of all the items of each scale.

#### The Chinese version of the Pedestrian Behavior Scale (CPBS)

The Pedestrian Behavior Scale (PBS) is a questionnaire to identify pedestrian behaviors toward other road users, developed and validated by Granié et al. [21]. In this study, pedestrian behaviors were assessed using the CPBS compiled by Qu et al. [26] (see S4 Appendix). This Chinese version is made up of 18 items selected from the French (40 items, four factors) and Turkish (13 items, three factors) PBS versions based on the four-factor structure, and translated into Chinese. Principle component analysis resulted in four dimensions: positive behaviors (5 items, *α* = .78), transgression (6 items, *α* = .72), aggressive behaviors (4 items, *α* = .64) and lapses (3 items, *α* = .61). Positive behaviors refer to the extent people act in the interest of others or are grateful for kindness from others when encountering other drivers, pedestrians, cyclists, etc. (e.g., “I thank a driver who stops to let me cross”). Transgression items include behaviors concerning offenses and errors (e.g., “I cross the street even when the pedestrian light is red”). Aggressive behaviors refer to the expression of negative emotions resulting in aggressive interactions between road users (e.g., “I become angry with other road users and insult them”). Lapses are related to lack of concentration on a task (e.g., “I forget to look both ways before crossing when I am thinking about something else”). Each item was answered on a 5-point Likert scale, ranging from 1 (“never”) to 5 (“always”), with higher scores denoting higher frequencies. We calculated a total score for each factor by adding the scores for all the items belonging to each factor.

#### Perceived Stress Scale-10 (PSS-10)

PSS-10 [66] is the 10-item version of the Perceived Stress Scale (PSS), which was primarily a 14-item tool developed by Cohen et al. [67] to measure the global level (opposite to event-specific level) of perceived stress. It measures the degree to which life situations are appraised as stressful. The PSS has become one of the most widely used instruments for measuring nonspecific global stress [68–72]. Here we used the Simplified Chinese version of PSS-10 translated by Wang et al. [73] (see S5 Appendix), with sufficient internal consistency (*α* = .86). Each question was answered on a 5-point Likert scale ranging from 0 (“never”) to 4 (“very often”), indicating how often participants have felt or thought in a certain way within the past month. A final PSS score was derived by adding the scores for all the 10 items.

#### Socio-demographics

Several sociodemographic variables were measured, including gender, age, education level, marital status, and work status.

### Procedure

This survey was conducted in Anshun, Guizhou. Several trained research interns, consisting of a group of selected local college students, distributed the paper questionnaires to residents in office buildings, corner shops, in front of supermarket gates, and houses, on a one to one basis. The survey was conducted on continuous days, including workdays and weekends, in spite of the effect of villainous weather or other special events. Adult males and females who were sound in mind and limb and able to read and answer questions were our potential investigation objects. They participated in this study voluntarily, and were assured that their information would be kept confidential and used only for scientific purpose. All residents were told that they just needed to complete the questionnaires based on own thoughts and behaviors. All questionnaires were completed in approximately 30 min. The participants returned them to the research interns after completion. The participants received CNY 50 (approximately USD 8) after completing the questionnaires.

### Analysis plan

All the data of our study is available (see S6 Appendix). To analyze the data, we carried out the following analyses:

a We first conducted descriptive statistics (the range, mean and standard deviation) and examined the internal consistency reliability of each scale. All missing values were replaced by the mean value of the indicated variable.
b Pearson correlation analysis was conducted to preliminarily explore the linear relations among personality variables, global perceived stress and pedestrian behavior variables. The statistic “*r*” was the correlation coefficient of two variables, reflecting the direction and the extent to which the two variables are linearly correlated. Generally in psychology, *r* = .10~.29 represents weak correlation, *r* = .30~.49 represents middle correlation, and *r* ≥ .50 means strong correlation. However, when the sample size is large enough (n ≥ 100), even *r* = .30 has strong statistical power. Whether correlation relationship exits is based on the result of significant testing (H0: *ρ* = 0). These analyses were conducted using the IBM SPSS v16.0 program.
c Multiple regression analysis was conducted to further determine the predictive value of the personality variables and stress on each type of pedestrian behavior. The general purpose of multiple regression is to learn more about the relationship between several independent or predictor variables and a dependent or criterion variable. It has been widely used in the social and natural sciences, which allows the researcher to ask (and hopefully answer) the general question "what is the best predictor of. . .". As is evident in the name multiple *linear* regression, it is assumed that the relationship between variables is linear. In general then, multiple regression procedures will estimate a linear equation of the form:

$$y=b_{1}x_{1}+b_{2}x_{2}+\ldots +b_{n}x_{n}+c+e$$

In this equation, *b*_*n*_ is the regression coefficient (or *B* coefficient) of the independent variable *x*_n_, representing the independent contribution of *x*_*n*_ to the prediction of the dependent variable (i.e., the contribution after controlling for all other independent variables). The constant term *c* is the y-intercept of the equation. And the error term *e* represents those unobservable factors that may affect the dependent variable. *R*^2^ (*R* Square), also known as the Coefficient of determination is a commonly used statistic to evaluate model fit, which reflects the extent to which the regression model can explain the variability of the dependent variable. Significant testing of the regression equation is conducted to examine whether the linear relation between the dependent variable and the independent variables reaches significance level. And significant testing of the regression coefficients is conducted to examine whether the effect of each independent variable on the dependent variable reaches significance level.

In our analyses, the hierarchical fashion of the regression model was adopted for the purpose of determining the extra contribution of the personality variables while controlling for age and gender, and the extra contribution of stress while controlling for age, gender and personality variables. Age and gender were controlled because gender differences have been generally discovered among the behaviors of road users such as pedestrians, cyclists and drivers [74], with significant gender and age effects on certain PBS dimensions reported [21]. Hierarchical multiple regression has been widely used to test whether one predictor variable contribute unique explanations of variance for the dependent variable, even confined to the traffic safety, health or related domains [26, 43, 55, 60, 75, 76]. Specifically, when establishing the model, age and gender were entered at step 1; personality variables were entered at step 2; and the PSS score was entered at step 3. These analyses were conducted using the IBM SPSS v16.0 program.

d Additionally, we used path analysis to further examine the hypothetic potential mediating effect of global perceived stress on the associations of personality traits with pedestrian behavior. Path analysis, one type of structural equation model, has been used to decompose a causal relationship into potential direct and indirect effects [77, 78] and has been reported in a similar study which explored the relationship among personality traits, driving-related anger and aggressive behavior in traffic [76]. We analyzed the covariance matrix using the IBM SPSS AMOS v21.0 program. Various fit indices were used to assess the fit of the model: *χ*^2^/*df* ratio, goodness-of-fit index (GFI), adjusted goodness-of-fit (AGFI), comparative fit index (CFI) and root-mean-square error of approximation (RMSEA). Good (acceptable) model fit is reflected by a *χ*^2^/*df* ratio less than 2 [79]. The GFI, AGFI, and CFI values exceeding .90 provide evidence that the model is a very good fit for the data [80, 81], and the RMSEA value below .06 is taken as representing a good fit [82, 83].

## Results

### Descriptive statistics

**Fig 1:**
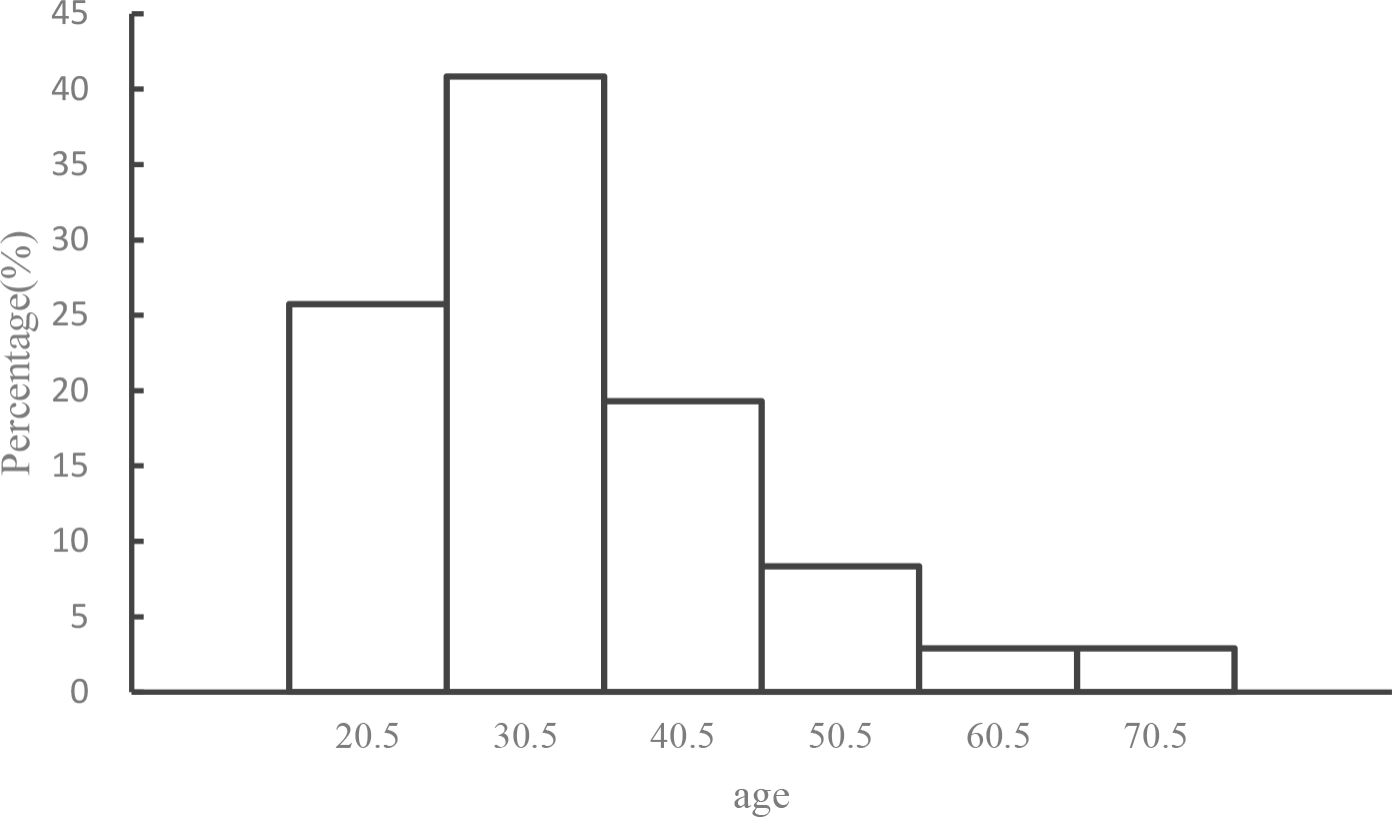
Age distribution histogram of all the 311 participants.

Among all the 311 participants, the ratio of men versus women was 143 (45.98%) to 166 (53.38%) (gender of two participants were unknown). The descriptive statistics for other socio-demographic variables (i.e., age and years of education) and each scale are shown in Table 1. The distribution of age for all the 311 participants is shown in Fig 1. Reliability was unsatisfactory for extraversion and normlessness because the Cronbach’s alpha was under .60, so these two scales were not included in subsequent analyses. Although the reliability of lapses was also not very acceptable (*α* = .49), we still decided to include it into subsequent analyses considering that it only has three items.

**Table 1:** Descriptive statistics for socio-demographic variables and each scale.

Variables	Items	Mean	SD	Range	*α*
Age	–	33.64	11.59	(16, 75)	–
Years of education	–	12.82	3.16	(2, 19)	–
Personality					
Extraversion	8	25.16	4.15	(12.00, 37.63)	.44
Openness	10	32.64	5.28	(13.00, 50.00)	.64
Neuroticism	8	20.83	5.02	(9.00, 38.00)	.65
Normlessness	4	8.90	2.77	(4.00, 17.00)	.41
Altruism	10	39.67	5.10	(20.00, 50.00)	.71
PSS	10	26.19	4.87	(10.00, 40.00)	.69
PBS					
Positive behaviors	5	17.88	3.98	(7.00, 25.00)	.70
Transgression	6	11.21	3.65	(6.00, 25.00)	.71
Aggressive behaviors	4	5.75	2.47	(4.00, 16.00)	.77
Lapses	3	5.84	2.05	(3.00, 15.00)	.49

*Notes*: PSS = Perceived Stress Scale; PBS = Pedestrian Behavior Scale.

### Relationships of personality, global stress and pedestrian behavior

#### Correlation analyses

Table 2 provides the Pearson correlation coefficients among age, gender, and all scale and subscale scores. There were various directions and extent to which each personality trait was correlated with a specific pedestrian behavior dimension. Neuroticism showed a significant negative correlation with positive behaviors (*r* = −.30, *p* < .001), and positively correlated with the other three items, i.e., transgression (*r* = .31, *p*< .001), aggressive behaviors (*r* = .27, *p* < .001) and lapses (*r* = .26, *p* < .001). The relationship between altruism and pedestrian behavior showed an opposite pattern. Openness showed a significant positive correlation with positive behaviors (*r* = .31, *p* < .001) and negative correlation with transgression (*r* = −.12, *p* < .05). Global stress had significant positive correlations with neuroticism (*r* = .55, *p* < .001), and negatively correlated with openness (*r* = −.21, *p* < .001) and altruism (*r* = −.26, *p* < .001). Global stress was also significantly correlated with all four pedestrian behavior dimensions, with a negative correlation with positive behaviors (*r* = −.26, *p* < .001) and positive correlations with the other three dimensions (*p* < .01).

**Table 2:** Correlations among age, gender, personality traits, global stress, and pedestrian behaviors.

Variables	1	2	3	4	5	6	7	8	9
1. Age	−								
2. Gender	−.02	−							
3. Openness	−.02	−.09	−						
4. Neuroticism	−.24***	.17**	−.29***	−					
5. Altruism	.14*	.09	.42***	−.45***	−				
6. PSS	−.17**	.14*	−.21***	.55***	−.26***	−			
7. Positive behaviors	.13*	−.13*	.31***	−.30***	.45***	−.26***	−		
8. Transgression	−.20***	−.06	−.12*	.31***	−.37***	.17**	−.21***	−	
9. Aggressive behaviors	−.10	−.07	−.09	.27***	−.45***	.18**	−.41***	.57***	−
10. Lapses	−.17**	.07	−.07	.26***	−.23***	.21***	−.18**	.54***	.44***

*Notes*: All tests are two-tailed. PSS = Perceived Stress Scale; Gender (1 = male, 2 = female).

^*^ *p* < .05 (two-tailed)

** *p* < .01 (two-tailed)

*** *p* < .001 (two-tailed).

#### Hierarchical multiple regression analyses

Results of the regression analyses are shown in Table 3. The *β* (Beta) value is the standardized coefficient of each independent variable.

**Table 3:** Regression of pedestrian behaviors on age, gender, personality traits, and global stress.

Predictors (*β*)		Dependent variables			
		Positive behaviors	Transgression	Aggressive behaviors	Lapses
**Step1**					
1	Age	.12*	−.19**	−.09	−.16**
2	Gender	−.13*	−.06	−.07	.06
	*R*^2^	.03	.04	.01	.03
	*ΔR*^2^	.03**	.04**	.01	.03**
	*F*	5.22**	6.48**	1.93	4.91**
**Step 2**					
1	Age	.06	−.11*	.00	−.10
2	Gender	−.14**	−.06	−.04	.06
4	Openness	.12*	.04	.12*	.05
5	Neuroticism	−.07	.17**	.10	.15*
7	Altruism	.38***	−.28***	−.44***	−.17*
	*R*^2^	.26	.18	.21	.09
	*ΔR*^2^	.22***	.14***	.20***	.06***
	*F*	20.92***	13.03***	16.54***	6.26***
**Step 3**					
1	Age	.05	−.11*	.00	−.10
2	Gender	−.14**	−.06	−.04	.05
4	Openness	.11*	.04	.12*	.05
5	Neuroticism	−.01	.17*	.08	.10
7	Altruism	.38***	−.28***	−.44***	−.17*
8	PSS	−.12*	.00	.04	.09
	*R*^2^	.27	.18	.22	.10
	*ΔR*^2^	.01*	.00	.00	.01
	*F*	18.24***	10.82***	13.84***	5.59***

*Notes*: PSS = Perceive Stress Scale; Gender (1 = male, 2 = female).

^*^ *p* < .05 (two-tailed)

** *p* < .01 (two-tailed)

*** *p* < .001 (two-tailed).

As shown in Table 3, age and gender entered the regression model in the first step and explained 3%, 4%, 1% and 3% of the variance in the four dimensions of pedestrian behavior respectively. In step 2, personality variables were added and explained an additional 22%, 14%, 20% and 6% of the variance in four behavior dimensions, respectively, and the unique contribution of each personality variable while controlling for age and gender was reflected by the *β* (Beta) value. As shown, altruism significantly contributed to positive behaviors in a positive direction (*β* = .38, *p* < .001), and to the other three in negative directions. Openness significantly contributed to positive behaviors (*β* = .12, *p* < .05) and aggressive behaviors (*β* = .12, *p* < .05) to both positive directions. Meanwhile, neuroticism significantly contributed to transgression (*β* = .17, *p* < .01) and lapses (*β* = .15, *p* < .05), to both positive directions. In step 3, PSS only explained an additional 1% of the variance in positive behaviors (*ΔR*^2^ = .01, *p* < .05), and significantly contributed to positive behaviors (*β* = .17, *p* < .01). It should be noted that after PSS was entered into the model, the significant effect of neuroticism on lapses disappeared, while the impact on transgression decreased. These data suggested that global stress may be a mediator of the relationship between neuroticism and pedestrian behaviors.

#### Path analysis

**Fig 2:**
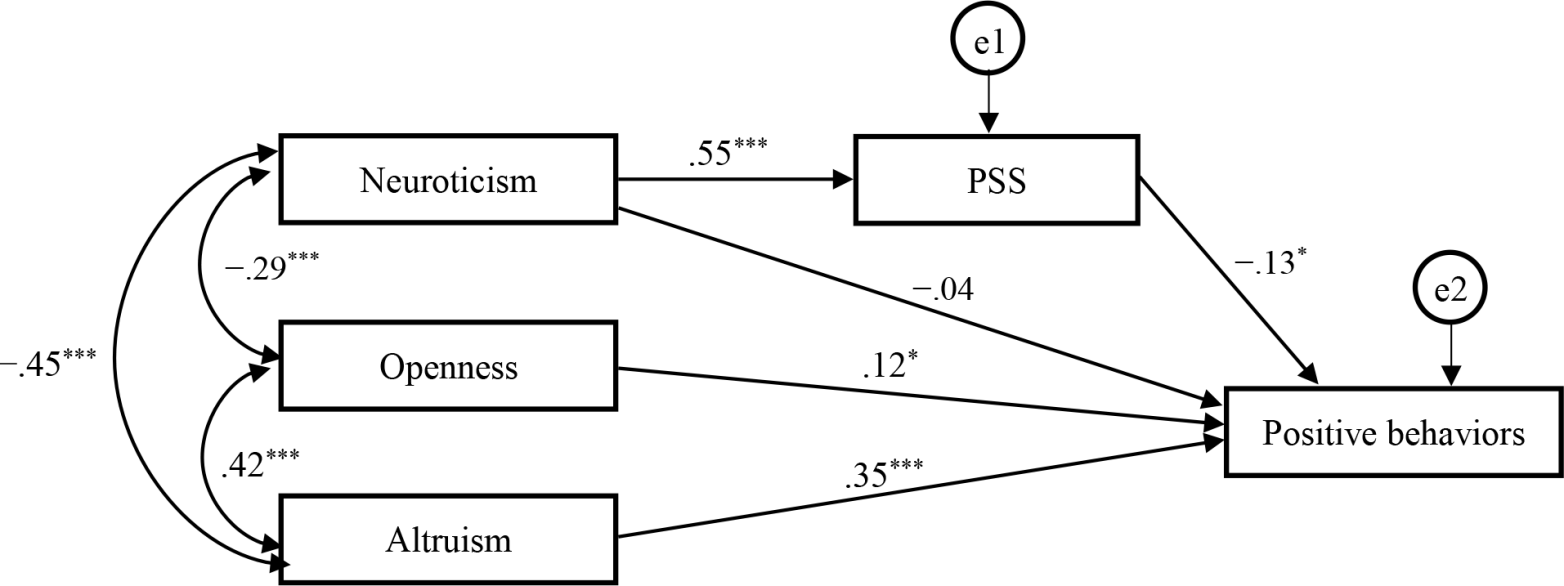
Final path model for personality traits, global stress and positive pedestrian behaviors. Path coefficients are standardized regression coefficients. ^*^ *p* < .05 (two-tailed), *** *p* < .001 (two-tailed).

To examine the potential mediating effect of global stress on the relationship between neuroticism and pedestrian behaviors, a path analysis was conducted. Since direct effects of PSS on the four pedestrian behavior dimensions except for positive behaviors were not significant, we only conducted a path model for positive behaviors. In the initial model, openness, neuroticism and altruism served as independent variables, while positive behaviors served as the dependent variable and PSS considered a mediating variable for the relationships between independent and dependent variables. The final model (Fig 2.) fit the data well (*χ*^2^ (2) = .98, *p* = .61, GFI = .999, AGFI = .991, CFI = 1.000, RMSEA = .000). Standardized regression coefficients for all pathways are presented. It was revealed that, the effects of neuroticism on positive behaviors seemed to be fully mediated by global stress. Higher neuroticism aroused more global stress, which then contributed to less positive behaviors. Openness and altruism directly contributed to positive behaviors.

## Discussion

This study explored the joint effect of five personality traits and global perceived stress on pedestrian behaviors in a Chinese sample. The CPBS was used to measure pedestrian behavior, and showed acceptable reliability except for the lapses subscale. Personality traits, including altruism, openness and neuroticism significantly affected different pedestrian behavior dimensions, and global perceived stress was also a significant predictor of positive pedestrian behaviors. In addition, it seems that the relation between trait neuroticism and positive pedestrian behaviors was completely mediated by global perceived stress.

Despite of the two personality traits we didn’t analyze because of low reliability, the other three (i.e., openness, neuroticism and altruism) all showed significant effects on pedestrian behavior. First, altruism significantly predicted all four pedestrian behavior dimensions, including positive behaviors, transgression, aggressive behaviors and lapses. In a prior study by our team, it was positive behaviors and lapses that altruism could significantly predict [26]. It seems that the predictive contribution of altruism on positive pedestrian behaviors and lapses are more stable. Individuals more concerned about others tend to act in the interest of others, and are grateful for kindness from other road users, thus exhibiting more positive behaviors. Such a positive climate may then help reduce errors and lapses caused by lack of concentration or negative emotional factors. This finding corroborated previous reports assessing traffic safety behaviors, which demonstrated that altruism negatively affects dangerous driving behaviors [55, 84, 85], and constitutes an effective predictor of traffic violations in China [27].

What we are more interested in is the results of openness and neuroticism, two dimensions of the Big Five Model of personality. As shown above, openness was found to be a significant predictor of positive and aggressive behaviors, interestingly, both in positive directions. That is to say, a pedestrian with high scores on the openness trait may exhibit more positive behaviors (e.g., thanking drivers who let him/her go first) and aggressive behaviors (e.g., becoming angry with other road users) at the same time. However, another study evaluating young adult pedestrians found no significant correlation between openness to experience and risky pedestrian behavior [22]. Although openness is strongly related to sensation seeking [37, 86], a trait positively correlated with risky behaviors [43], the direct effect of openness to risky behaviors remains uncertain. It is noteworthy that, there were also contradictory studies which have revealed that both higher and lower scores of openness are correlated with more dangerous driving, with openness positively correlated with constructive behaviors [75, 87], in line with the current results, to some extent. One possible explanation is that both good and bad actions, relevant to normal actions, are defined as a new and unusual experience. Thus pedestrians who scored high in openness tend to have more “unusual experience” like positive or aggressive behaviors. Therefore, it seems that the contribution of openness to behavior in the traffic context is somewhat sophisticated and needs further exploration.

Neuroticism was a significant predictor of transgression and lapses, in line with previous studies assessing the behavior of road users and accidents, with neuroticism showing positive correlations with aggressive driving [76], vehicular accidents [33] and road rage [45, 46]. High neuroticism means more inclination to experience negative emotions and difficulty in dealing with problems [76]; this leads to inappropriate coping with events in the traffic context. It was demonstrated that individuals with high neuroticism scores are often impatient, anxious, tense, and irritated [88], with greater impulsiveness [89]. All these factors may contribute to more negative behaviors like transgression and lapses as a pedestrian.

Global perceived stress was significantly correlated with all four pedestrian behavior dimensions, and an effective predictor of positive behaviors after controlling for personality traits, although this effect was not very strong (*β* = −.12). Pedestrians with lower global stress levels may behave more positively. This provided more evidence to the notion that stress may be an important factor influencing road safety, given that both global [55] and event-specific [90–92] levels of perceived stress affect driving safety. Our finding also suggested that despite negative behaviors such as aggression and errors on the road, stress may also have something to do with positive behavioral aspects, which has not been reported much before. Since positive behaviors usually request some extra actions (e.g., expression of gratitude to other road users), it is possible that the limited cognitive resource of working memory is overloaded by worrisome thoughts, which are activated in stressful situations [93], thus not sufficient for extra positive behaviors.

Another main finding of the current study is that global stress fully mediated the effect of trait neuroticism on positive pedestrian behaviors. That is, unfriendly performance for individuals with high neuroticism scores depended upon the extent to which global stress in life is perceived. In fact, numerous studies have strongly linked trait neuroticism with stress perception and responses [57, 60, 76, 94, 95]. For example, neurotic individuals have reported more occurrence of daily stressors [57]. High neuroticism also impairs stress responses, including more negative appraisals, lower positive affection and higher negative affection, and less efficient coping strategies [57, 76, 94]. This does not only increase the cognitive load, but also arouses negative emotions, which consequently diminish kind or grateful reactions to other road users as pedestrians. This mediating effect helped us deeper understand individual differences (e.g., personality) which generally appears in various behaviors (e.g., pedestrian behavior), since it seems more feasible to build models around specific processes from stress perception to the final performance than analyzing the relation between personality, a basic reaction tendency to the environment, and realistic behaviors.

The results also showed significant effects of socio-demographic variables (i.e., age and gender) on pedestrian behavior. Specifically, younger people committed more transgression. This result was consistent with previous study [21]. This may be due to the well-known high risk-taking tendency during adolescence. However, in our study, the male showed more positive behaviors than the female, opposite to the previous studies [21, 26]. In some other studies, men also committed more risky pedestrian behaviors like violations [96] and shorter waiting time at signalized crosswalk [97], and accounted for 70% of pedestrian death [98]. We suspected that this could be partially explained by the level of education of the two groups, since years of education of women was marginally significantly fewer than that of men (*t* (303.97) = 1.72, *p* = .087), and years of education was significantly positively correlated with positive behaviors (*r* = .27, *p* < .001) and negatively correlated with aggressive behaviors (*r* = −.12, *p* < .05). However, when years of education was controlled by entering the regression model, the coefficient of gender was still significantly negative (*β* = −.13, *p* < .05), meaning that women committed less positive behaviors than men when excluding the effect of education level. Another possible reason is that the gender role of women in the social environment of Anshun is different from general feminine stereotypes such as encouraging mutual assistance and caring for others, especially after experiencing an urbanization process. This explanation, however, needs to be further confirmed in future studies.

Some limitations of this study should be mentioned. First, participants were selected through convenience sampling, and the sample may not exactly represent the national pedestrian population. Indeed, participants were not selected according to population composition for various characteristics (e.g., gender, age and districts). Therefore, whether our conclusions can be generalized to the entire population of pedestrians in China is questionable. Another limitation is the self-reporting method on which data acquisition was based. Although self-reports are commonly used in such studies, social desirability bias or similarity of expression remain inevitable. Future work would consider using other measurements like field observations or simulated tasks to supplement self-reports. Finally, the reliabilities of three scales (i.e., extraversion, normlessness and lapses of the CPBS) were unsatisfactory. Although the BFI-44 has been widely used in studies based on Chinese samples (e.g., [99, 100, 55]), these studies rarely reported the internal consistency reliability of the extraversion subscale. A recent cross-cultural study showed the instrument had acceptable reliability across 56 nations in 10 world regions including Hong Kong (China) [101], and another study examined the psychometric attributes of a Chinese-language BFI-44, showing that the internal reliability of extraversion was good (*α* > .70). We suspected that the low reliability in this study may be partly due to regional discrepancy, since our sample only come from one city: Anshun. However, it needs to be further verified. As to normlessness, we found it’s internal reliability unsatisfactory in a previous work of ours (*α* < .60) [55], while another Chinese study reported an acceptable reliability (*α* > .60) [27]. In consideration that coefficient alpha is influenced by the number of items in a scale [102, 103], the low reliability of normlessness (4 items) and lapses (3 items) of CPBS may be partly explained. Even so, we tended to believe that the rest of the results was receivable, since dimensions of a specific scale is mutually independent from each other.

In conclusion, this study mainly focused on the joint effect of individual variables (personality and stress) that may affect pedestrian behavior, as a supplement to environmental or social factors. Using a sample of 311 subjects, we found that altruism, neuroticism and openness effectively predicted several pedestrian dimensions, in both positive and negative aspects. The global level of perceived stress also predicted positive behaviors and mediated the relation between neuroticism and positive behaviors. These findings increase awareness of stress bringing extensive damage in daily life, especially among individuals with certain characteristics (e.g., high neuroticism). Even basic and relatively easy activities like walking on the street can be seriously affected. These results may also help identify individuals who are prone to accident. In dealing with this problem, methods for coping with stress, e.g. Mindfulness-Based Stress Reduction [104], may help reduce road accidents caused by the improper behaviors of pedestrians and drivers.

## Supporting information

S1 Appendix The Big Five Inventory-44 (BFI-44). (DOCX)

S2 Appendix The normlessness items. (DOCX)

S3 Appendix The altruism items. (DOCX)

S4 Appendix The Chinese version of the Pedestrian Behavior Scale (CPBS). (DOCX)

S5 Appendix The Perceived Stress Scale-10 (PSS-10). (DOCX)

S6 Appendix Data. (XLSX)
