# Supplementary material for: The joint effect of personality traits and perceived stress on pedestrian behavior in a Chinese sample
Source: PLoS One. 2017 Nov 30;12(11):e0188153. doi: 10.1371/journal.pone.0188153 (PMC5708679; doi:10.1371/journal.pone.0188153)
Supplement: S1 Appendix — (DOCX) [file pone.0188153.s001.docx]

**The Big Five Inventory-44 (BFI-44)**

Here are a number of characteristics that may or may not apply to you. For example, do you agree that you are someone who likes to spend time with others? Please write a number next to each statement to indicate the extent to which you agree or disagree with that statement.

1 = Strongly disagree, 2 = Disagree a little, 3 = Neither agree nor disagree,

4 = Agree a little, 5 = Strongly agree

I see Myself as Someone Who...

| ___1. Is talkative | ___23. Tends to be lazy |
| --- | --- |
| ___2. Tends to find fault with others | ___24. Is emotionally stable, not easily upset |
| ___3. Does a thorough job | ___25. Is inventive |
| ___4. Is depressed, blue | ___26. Has an assertive personality |
| ___5. Is original, comes up with new ideas | ___27. Can be cold and aloof |
| ___6. Is reserved | ___28. Perseveres until the task is finished |
| ___7. Is helpful and unselfish with others | ___29. Can be moody |
| ___8. Can be somewhat careless | ___30. Values artistic, aesthetic experiences |
| ___9. Is relaxed, handles stress well | ___31. Is sometimes shy, inhibited |
| ___10. Is curious about many different things | ___32. Is considerate and kind to almost everyone |
| ___11. Is full of energy | ___33. Does things efficiently |
| ___12. Starts quarrels with others | ___34. Remains calm in tense situations |
| ___13. Is a reliable worker | ___35. Prefers work that is routine |
| ___14. Can be tense | ___36. Is outgoing, sociable |
| ___15. Is ingenious, a deep thinker | ___37. Is sometimes rude to others |
| ___16. Generates a lot of enthusiasm | ___38. Makes plans and follows through with them |
| ___17. Has a forgiving nature | ___39. Gets nervous easily |
| ___18. Tends to be disorganized | ___40. Likes to reflect, play with ideas |
| ___19. Worries a lot | ___41. Has few artistic interests |
| ___20. Has an active imagination | ___42. Likes to cooperate with others |
| ___21. Tends to be quiet | ___43. Is easily distracted |
| ___22. Is generally trusting | ___44. Is sophisticated in art, music, or literature |

Please check: Did you write a number in front of each statement?

BFI scale scoring (“R” denotes reverse-scored items):

**Extraversion: 1, 6R, 11, 16, 21R, 26, 31R, 36 (we used)**

Agreeableness: 2R, 7, 12R, 17, 22, 27R, 32, 37R, 42 (we didn’t use)

Conscientiousness: 3, 8R, 13, 18R, 23R, 28, 33, 38, 43R (we didn’t use)

**Neuroticism: 4, 9R, 14, 19, 24R, 29, 34R, 39 (we used)**

**Openness: 5, 10, 15, 20, 25, 30, 35R, 40, 41R, 44 (we used)**
