# Supplementary material for: The joint effect of personality traits and perceived stress on pedestrian behavior in a Chinese sample
Source: PLoS One. 2017 Nov 30;12(11):e0188153. doi: 10.1371/journal.pone.0188153 (PMC5708679; doi:10.1371/journal.pone.0188153)
Supplement: S2 Appendix — (DOCX) [file pone.0188153.s002.docx]

**The Normlessness items**

Here are a number of descriptions that you may or may not agree with. Please write a number next to each statement to indicate the extent to which you agree or disagree with that statement.

1 = Strongly disagree, 2 = Disagree a little, 3 = Neither agree nor disagree,

4 = Agree a little, 5 = Strongly agree

| ___1. It is all right to do anything you want as long as you keep out of trouble. |
| --- |
| ___2. It is OK to get round laws and rules as long as you don’t break them directly. |
| ___3. If something works, it is less important whether it is right or wrong. |
| ___4. Some things can be wrong to do even though it is legal to do it. |
