# Supplementary material for: The joint effect of personality traits and perceived stress on pedestrian behavior in a Chinese sample
Source: PLoS One. 2017 Nov 30;12(11):e0188153. doi: 10.1371/journal.pone.0188153 (PMC5708679; doi:10.1371/journal.pone.0188153)
Supplement: S3 Appendix — (DOCX) [file pone.0188153.s003.docx]

**The Altruism items**

Here are a number of characteristics that may or may not apply to you. Please write a number next to each statement to indicate the extent to which you agree or disagree with that statement.

1 = Strongly disagree, 2 = Disagree a little, 3 = Neither agree nor disagree,

4 = Agree a little, 5 = Strongly agree

I see Myself as Someone Who...

| ___1. Make people feel welcome. |
| --- |
| ___2. Anticipate the needs of others. |
| ___3. Love to help others. |
| ___4. Am concerned about others. |
| ___5. Have a good word for everyone. |
| ___6. Look down on others. |
| ___7. Am indifferent to the feelings of others. |
| ___8. Make people feel uncomfortable. |
| ___9. Turn my back on others. |
| ___10. Take no time for others. |
