# Supplementary material for: The joint effect of personality traits and perceived stress on pedestrian behavior in a Chinese sample
Source: PLoS One. 2017 Nov 30;12(11):e0188153. doi: 10.1371/journal.pone.0188153 (PMC5708679; doi:10.1371/journal.pone.0188153)
Supplement: S4 Appendix — (DOCX) [file pone.0188153.s004.docx]

**The Chinese version of the Pedestrian Behavior Scale (CPBS)**

Below we will ask about your feelings or thoughts about some events in the past month. Some of these questions may seem similar on the surface, but they are actually different and should be treated differently. It is best not to try to calculate the exact number of times, but give a realistic estimate as soon as possible. Please select one of the five choices in each of the following questions as your answer and play "√" in the corresponding box.

1 = Never, 2 = Very few, 3 = Sometimes, 4 = Often, 5 = Very often

| Items | 1 | 2 | 3 | 4 | 5 |
| --- | --- | --- | --- | --- | --- |
| 1. I walk on the right-hand side of the sidewalk so as not to bother the pedestrians I meet. |  |  |  |  |  |
| 2. I thank a driver who stops to let me cross. |  |  |  |  |  |
| 3. When I am accompanied by other pedestrians, I walk in single file on narrow sidewalks so as not to bother the pedestrians I meet. |  |  |  |  |  |
| 4. I stop to let the pedestrians I meet by. |  |  |  |  |  |
| 5. I let a car go by, even if I have the right-of-way, if there is no other vehicle behind it. |  |  |  |  |  |
| 6. I cross between vehicles stopped on the roadway in traffic jams. |  |  |  |  |  |
| 7. I cross without looking, following other people who are crossing. |  |  |  |  |  |
| 8. I realize that I do not remember the route I have just taken. |  |  |  |  |  |
| 9. I cross in between parked cars when there is a safer place to cross nearby. |  |  |  |  |  |
| 10. I cross while talking on my cell phone or listing to music on my headphones. |  |  |  |  |  |
| 11. I cross the street even though the pedestrian light is red. |  |  |  |  |  |
| 12. I cross even though the traffic light is still green for vehicles. |  |  |  |  |  |
| 13. I cross without looking because I am talking with someone. |  |  |  |  |  |
| 14. I cross outside the pedestrian crossing even if there is one less than 50 m away. |  |  |  |  |  |
| 15. I look at the traffic light and start crossing as soon as it turns red. |  |  |  |  |  |
| 16. I get angry with another user (pedestrian, driver, cyclist, etc.) and I make a hand gesture. |  |  |  |  |  |
| 17. I deliberately walk on the roadway when I could walk on the sidewalk or on the shoulder. |  |  |  |  |  |
| 18. I cross very slowly to annoy a driver. |  |  |  |  |  |
