# Supplementary material for: The joint effect of personality traits and perceived stress on pedestrian behavior in a Chinese sample
Source: PLoS One. 2017 Nov 30;12(11):e0188153. doi: 10.1371/journal.pone.0188153 (PMC5708679; doi:10.1371/journal.pone.0188153)
Supplement: S5 Appendix — (DOCX) [file pone.0188153.s005.docx]

**The Perceived Stress Scale-10 (PSS-10)**

Below we will ask about your feelings or thoughts about some events in the past month. Some of these questions may seem similar on the surface, but they are actually different and should be treated differently. It is best not to try to calculate the exact number of times, but give a realistic estimate as soon as possible. Please select one of the five choices in each of the following questions as your answer and play "√" in the corresponding box.

0 = Never, 1 = Very few, 2 = Sometimes, 3 = Often, 4 = Very often

| Items | 0 | 1 | 2 | 3 | 4 |
| --- | --- | --- | --- | --- | --- |
| 1. In the last month, how often have you been upset because of something that happened unexpectedly? |  |  |  |  |  |
| 2. In the last month, how often have you unable to control the important things in your life? |  |  |  |  |  |
| 3. In the last month, how often have you felt nervous and ‘stressed’? |  |  |  |  |  |
| 4. In the last month, how often have you confident about your ability to handle your personal problems? |  |  |  |  |  |
| 5. In the last month, how often have you felt that things were going your way? |  |  |  |  |  |
| 6. In the last month, how often have you found that you could not cope with all the things that you had to do? |  |  |  |  |  |
| 7. In the last month, how often have you been able to control irritations in your life? |  |  |  |  |  |
| 8. In the last month, how often have you felt that you were on top of things? |  |  |  |  |  |
| 9. In the last month, how often have you been angered because of things that were outside of your control? |  |  |  |  |  |
| 10. In the last month, how often have you felt difficulties were piling up so high that you could not overcome them? |  |  |  |  |  |
